# Hydroxychloroquine inhibits IL-1β production from amyloid-stimulated human neutrophils

**DOI:** 10.1186/s13075-019-2040-6

**Published:** 2019-11-27

**Authors:** Yuya Fujita, Naoki Matsuoka, Jumpei Temmoku, Makiko Yashiro Furuya, Tomoyuki Asano, Shuzo Sato, Hiroko Kobayashi, Hiroshi Watanabe, Eiji Suzuki, Takeshi Urano, Hideko Kozuru, Hiroshi Yatsuhashi, Tomohiro Koga, Atsushi Kawakami, Kiyoshi Migita

**Affiliations:** 1 Department of Rheumatology, Fukushima Medical University School of Medicine, 1 Hikarigaoka, Fukushima, Fukushima 960-1295 Japan; 2 Department of Rheumatology, Ohta Nishinouchi General Hospital Foundation, 2-5-20 Nishinouchi, Koriyama, Fukushima 963-8558 Japan; 3 Department of Biochemistry, Shimane University School of Medicine, Izumo, 693-8501 Japan; 4 Clinical Research Center, NHO Nagasaki Medical Center, Kubara 2-1001-1 Omura, Nagasaki, 856-8562 Japan; 5 Department of Immunology and Rheumatology, Unit of Advanced Preventive Medical Sciences, Nagasaki University Graduate School of Biomedical Sciences, 1-7-1 Sakamoto, Nagasaki, 852-8501 Japan

**Keywords:** Amyloid, Hydroxychloroquine, Inflammasome, Interleukin-1 beta, Neutrophils, NLR family pyrin domain containing 3, Serum amyloid A

## Abstract

**Background:**

Hydroxychloroquine (HCQ) is used for the treatment of patients with rheumatic diseases. We tested the hypothesis that HCQ affects the NLRP3 inflammasome, which is involved in autoinflammation.

**Methods:**

Human neutrophils were stimulated with serum amyloid A (SAA) in vitro and measured for IL-1β and caspase-1 (p20) secretion by ELISA. Pro-IL-1β mRNA expression in human neutrophils was quantified by real-time RT-PCR.

**Results:**

SAA stimulation induced significant production of IL-1β in human neutrophils. SAA stimulation also induced NF-κB activation, pro-IL-1β mRNA expression, and NLRP3 protein expression in human neutrophils. HCQ pretreatment significantly inhibited the SAA-induced IL-1β production in human neutrophils, but did not affect the SAA-induced NF-κB activation, pro-IL-1β mRNA expression, and NLRP3 protein expression. Furthermore, SAA stimulation induced cleaved caspase-1 (p20) secretion from human neutrophils, and this release was suppressed by HCQ pretreatment.

**Conclusions:**

Treatment with HCQ was associated with impaired production of IL-1β in SAA-stimulated human neutrophils without affecting the priming process of the NLRP3 inflammasome such as pro-IL-1β or NLRP3 induction. These findings suggest that HCQ affects the NLRP3 activation process, resulting in the impaired IL-1β production in human neutrophils, as representative innate immune cells.

## Background

Hydroxychloroquine (HCQ) exerts various anti-inflammatory and immunomodulatory effects and is widely used for the treatment of rheumatoid arthritis (RA) and systemic lupus erythematosus (SLE) [1]. However, its mode of action is not completely understood [2]. According to the previous studies, the actions of HCQ on the immune system appear to involve its ability to interfere with lysosomal acidification and thereby affect antigen processing and Toll-like receptor (TLR) signaling [2–4]. The NLRP3 inflammasome is a cytoplasmic macromolecular complex that orchestrates inflammatory responses in innate immunity by inducing caspase-1 activation and IL-1β processing [5]. Various danger signals, including reactive oxygen species (ROS), crystals, potassium efflux, and amyloid proteins, have been identified as possible activators of the NLRP3 inflammasome [6]. Recent studies demonstrated the pivotal roles of SAA in the regulation of immunity and inflammation. Cytokine-like activities of SAA for its induction of IL-1β, TNFα, and IL-8 had been demonstrated. The engagement of formyl peptide receptor-like 1 (FPRL1) by its ligand, SAA, initiates intracellular signaling such as nuclear factor-κB, leading to an activation of the innate immunity that is crucial to the development of inflammation [7]. Also, SAA has been identified as an endogenous activator of the NLRP3 inflammasome, which is critical for pro-IL-1β processing and activation [8]. Collectively, these findings illustrate proinflammatory functions of SAA including inflammasome activation.

Recent evidence suggests that the inflammasome is dysregulated in SLE and plays an important role in lupus-associated organ damage [9]. For example, NLRP3 inflammasome contributes to glomerular injuries and proteinuria in lupus nephritis [10]. Therefore, inflammasome-targeted therapies appear to be a new approach for lupus treatment. Because of the diverse effects of HCQ on pattern-recognition receptors [11], it is tempting to speculate that the inflammasome could be a target for HCQ during innate immune cell activation. On the basis of its high affinity for the lysosomal/endosomal compartment [3], we hypothesized that HCQ may affect the process for inflammasome activation. In the present study, we directly addressed the above hypothesis by determining the effects of HCQ on the NLRP3 inflammasome activation process using human neutrophils as representative innate immune cells. Our findings demonstrated that HCQ inhibited amyloid-mediated NLRP3 inflammasome activation and IL-1β secretion from human neutrophils. Taken together, the present findings provide novel insights toward understanding the anti-inflammatory effects of HCQ on the innate immune system.

## Materials and methods

### Reagents

Recombinant human SAA was purchased from Peprotech (Rocky Hills, NJ). The endotoxin levels were less than 0.1 ng/μg protein. Anti-β-actin antibodies were purchased from Santa Cruz Biotechnology Inc. (Dallas, USA). Anti-NLRP-3 antibody was purchased from MERCK MILLIPORE (Billerica, MA, USA). Human IL-1β and caspase-1 (p20) ELISA kits were purchased from R&D systems (Minneapolis, USA). Anti-phospho-NF-κB p65 (Ser536) antibody was purchased from Cell Signaling Technology (CST, Danvers, USA). Hydroxychloroquine, iberiotoxin (IBTX), lipopolysaccharide (LPS), and adenosine 5 triphosphate (ATP) were purchased from Sigma-Aldrich (Tokyo, Japan).

### Neutrophil isolation

Venous peripheral blood was obtained from Japanese healthy subjects (6 males, 1 females, mean age of 34.7 ± 7.8 years). Written informed consent for blood donation was obtained from each individual. The blood was layered on a Polymorphprep TM (Axis-Shield, Oslo, Norway) cushion, and cells were isolated according to the manufacturer’s protocol. Briefly, neutrophils were isolated on the basis of density, washed once in 0.5 N RPMI-1640 to restore osmolality, and then washed once more in RPMI-1640. Using this procedure, we obtained higher purity of CD13^+^ neutrophils. The cells were subsequently diluted in a complete medium consisting of RPMI-1640.

### ELISA and Western blot analysis

Neutrophils (2 × 10^6^/ml) were seeded in 24-well plates containing RPMI1640 supplemented with 10% heat-inactivated FBS and stimulated with SAA. Cell-free supernatants were collected by centrifugation at 400*g* for 5 min and assayed for IL-1β or caspase-1 (p20) using ELISA kits (R&D Systems, Minneapolis, MN, USA).

#### Cell lysis and Western blotting

Freshly isolated neutrophils were stimulated with SAA (10 μg/ml) for indicated periods, and the cells were washed by ice-cold PBS and lysed with RIPA Buffer (Sigma-Aldrich) supplemented with 1.0 mM sodium orthovanadate, 10 μg/mL aprotinin, and 10 μg/mL leupeptin for 20 min at 4 °C. After 5 min on ice, the cell lysates were centrifuged at 10,000*g* for 10 min at 4 °C. After centrifugation, cellular lysates (30 μg) were also subjected to 12% SDS-PAGE, followed by Western blot with antibodies against human NLRP3, phospho-NF-κB, or β-actin with an ECL Western blotting kit (Amersham, Little Chalfont, UK).

### Reverse transcription-polymerase chain reaction (RT-PCR)

Total RNA was extracted from neutrophils using the RNeasy total RNA isolation protocol (Qiagen, Crauley, UK) according to the manufacturer’s protocol. First-strand cDNA was synthesized from 1 μg of total cellular RNA using an RNA PCR kit (Takara Bio Inc., Otsu, Japan) with random primers. Thereafter, cDNA was amplified using specific primers respectively. The amplification of the IL-1β transcripts was also accomplished on a Light Cycler (Roche Diagnostics, Mannheim, Germany) using specific primers. The housekeeping gene fragment of glyceraldehydes-3-phosphates dehydrogenase (GAPDH) was used for verification of equal loading.

### Statistical analysis

Differences between groups were examined for statistical significance using the Student *t* test. *p* values less than 0.05 were considered statistically significant.

## Results

**Fig. 1:**
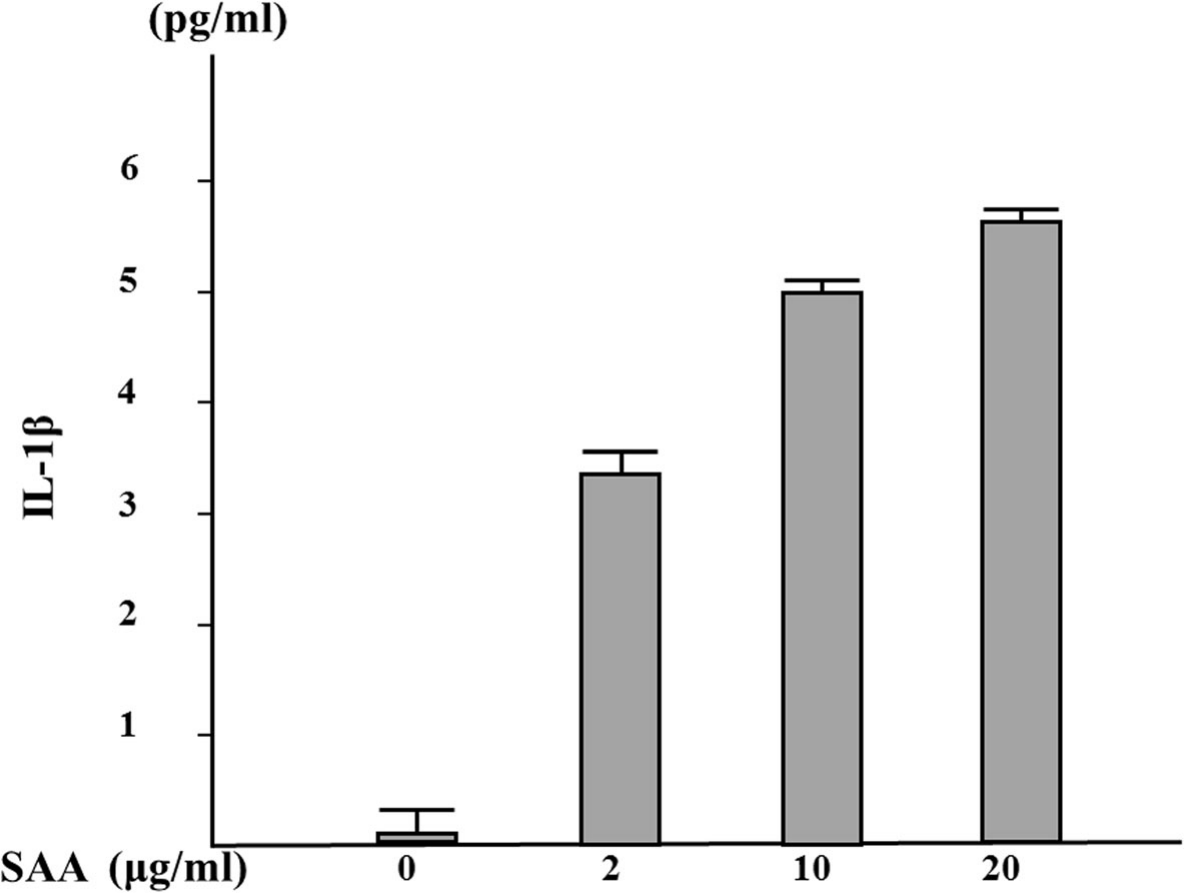
SAA induces IL-1β synthesis from neutrophils in a dose-dependent manner. Neutrophils (2 × 10^6^/ml) were incubated with the indicated concentrations of SAA for 24 h, and supernatants were analyzed for IL-1β production by ELISA. Values represent the mean ± SD of two independent experiments

**Fig. 2:**
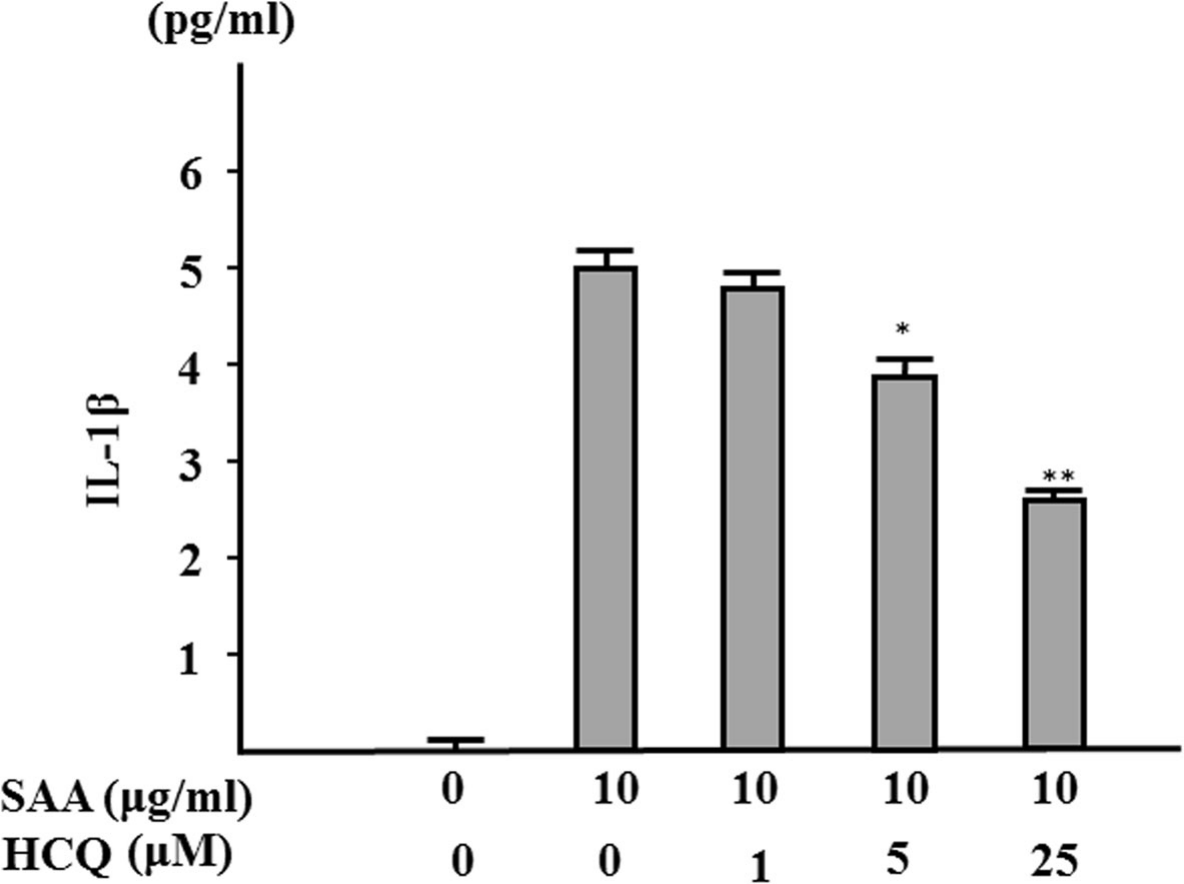
Hydroxychloroquine inhibits the IL-1β synthesis from SAA-stimulated neutrophils. Neutrophils were pretreated with the indicated concentrations of hydroxychloroquine for 1 h and stimulated with SAA (10 μg/ml) for 24 h, and supernatants were analyzed for IL-1β production by ELISA. Values represent the mean ± SD of two independent experiments. *p < 0.01 compared to SAA-stimulated neutrophils. **p < 0.001 compared to SAA-stimulated neutrophils

**Fig. 3:**
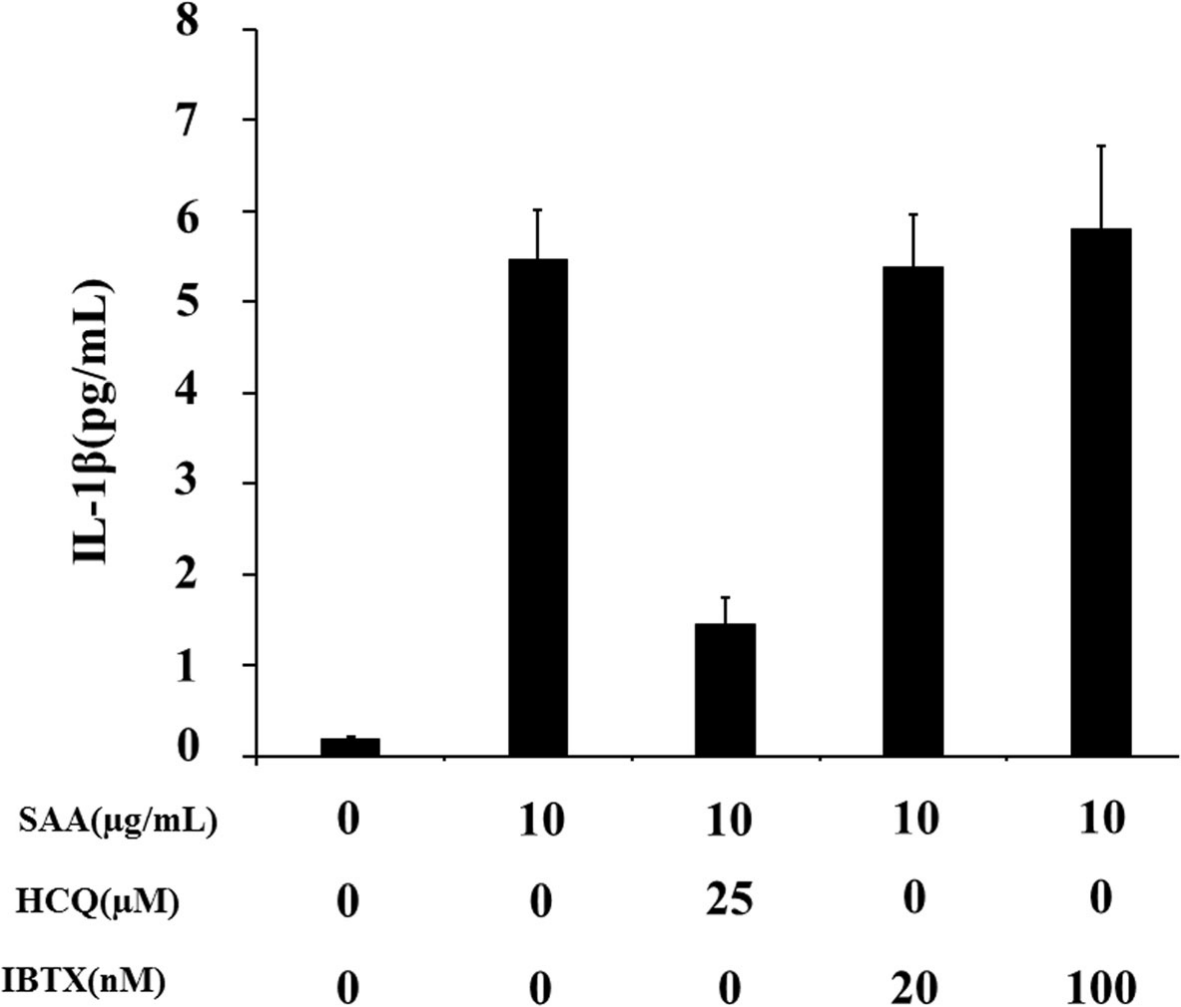
Hydroxychloroquine inhibits the IL-1β synthesis from SAA-stimulated neutrophils. Neutrophils were pretreated with the indicated concentrations of hydroxychloroquine or iberiotoxin (IBTX) for 1 h and stimulated with SAA (10 μg/ml) for 24 h, and supernatants were analyzed for IL-1β production by ELISA. Values represent the mean ± SD of two independent experiments

We previously showed that serum amyloid A (SSA) is capable of inducing IL-1β secretion from human neutrophils without a priming signal [12]. In this study, we investigated the effect of HCQ on SAA-induced NLRP3 inflammasome activation and subsequent IL-1β secretion in human neutrophils. As shown in Fig. 1, SSA stimulation alone induced IL-1β secretion from human neutrophils and reached a plateau at 10 μg/ml. Neutrophils were pretreated with various HCQ concentrations for 1 h and exposed to SAA (10 μg/ml). The supernatants were analyzed for their IL-1β contents by ELISA. HCQ pretreatment suppressed IL-1β secretion from SAA-stimulated neutrophils in a dose-dependent manner (Fig. 2). It was reported that ATP-induced K^+^ efflux plays a key role in activating the NLRP3 inflammasome and HCQ inhibits this inflammasome activation process by inhibiting Ca^++^-activated K^+^ channels (KCa) [13]. To address the involvement of Ca^++^-activated K^+^ channels, we examined the effects of iberiotoxin (IBTX), a specific Ca^++^-activated K^+^ channel inhibitor, against SAA-induced inflammasome activation. ATP-induced IL-1β secretion from LPS-primed neutrophils was blocked by IBTX as described previously [13] (data not shown), whereas IBRX pretreatment did not affect SAA-induced IL-1β secretion in neutrophils (Fig. 3), suggesting that KCa may not be involved in SAA-mediated inflammasome activation.

**Fig. 4:**
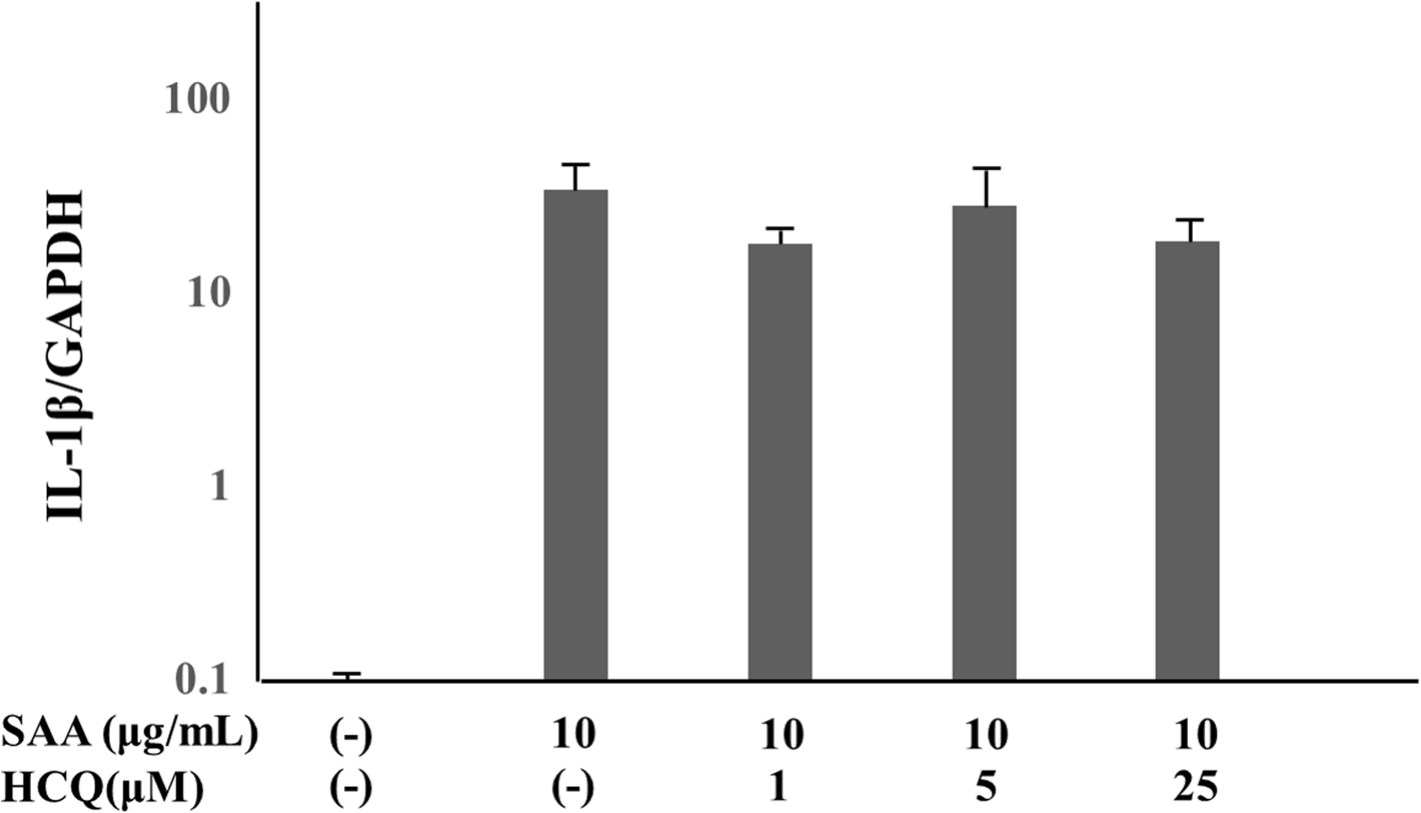
SAA induces the transcription of pro-IL-1β in human neutrophils. Neutrophils were pretreated with the indicated concentrations of hydroxychloroquine for 1 h and stimulated with SAA (10 μg/ml) for 8 h. The cells were harvested and analyzed for pro-IL-1β and GAPDH mRNA levels by real-time PCR. Values represent the mean ± SD of two independent experiments. *NS (not significant) compared to SAA-stimulated neutrophils

Next, we examined the effect of SAA on pro-IL-1β mRNA expression in human neutrophils. As shown in Fig. 4, SAA induced pro-IL-1β mRNA expression in these cells. HCQ pretreatment did not affect pro-IL-1β mRNA expression in SAA-stimulated neutrophils and SAA remained a potent inducer of pro-IL-1β mRNA expression.

**Fig. 5:**
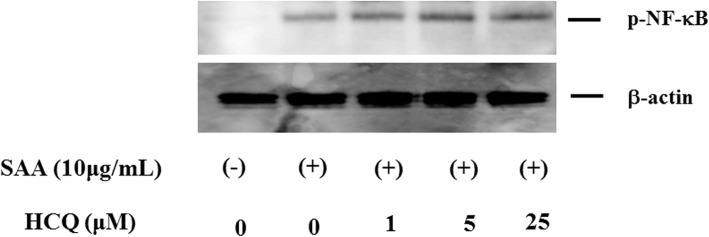
Phosphorylation of NF-κB p65 in SAA-treated neutrophils. Neutrophils were pretreated with the indicated concentrations of hydroxychloroquine for 2 h and stimulated with SAA (10 μg/ml) for 20 min. Cells were lysed and cellular lysates were subjected to Western blot using anti-phosphor-NF-κB and β-actin antibodies. Data are representative of two independent experiments

**Fig. 6:**
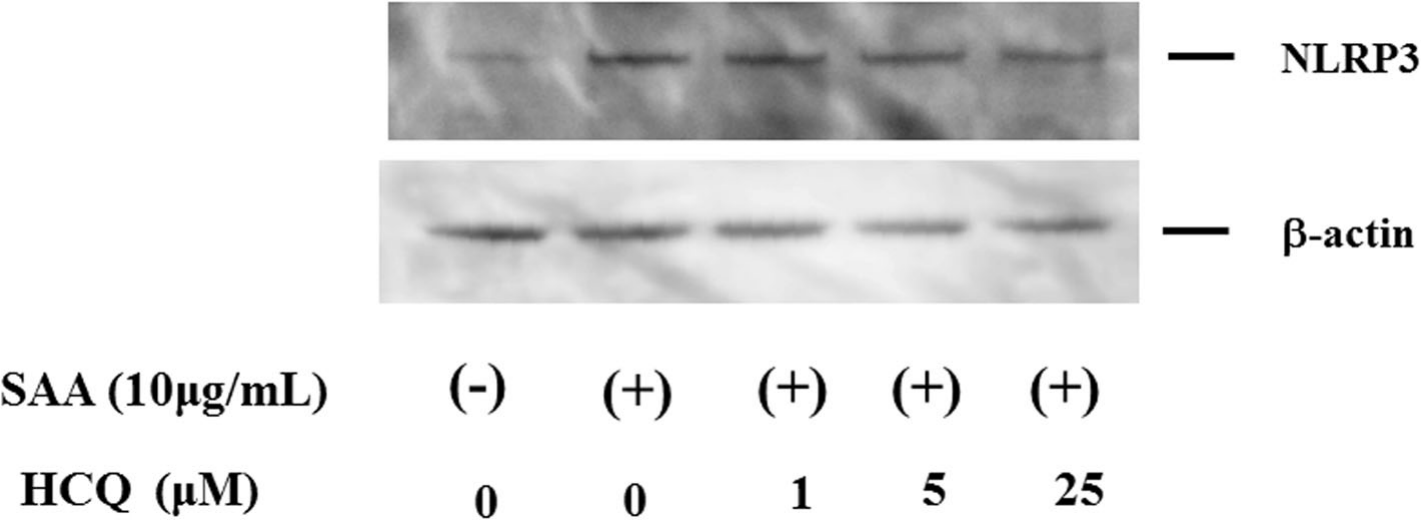
NLRP3 expression in neutrophils. Neutrophils were pretreated with the indicated concentrations of hydroxychloroquine for 1 h and stimulated with SAA (10 μg/ml) for 24 h. Cellular lysates were analyzed by Western using anti-NLRP3 or anti-β-actin antibodies. Three experiments were performed using different neutrophils, and a representative result is shown

As a key mediator of immunity, NF-κB plays a critical role in priming the NLRP3 inflammasome [14]. The NF-κB pathway is also involved in the transcription of pro-IL-1β [15]. Thus, we examined whether HCQ pretreatment (2 h) modulated the NF-κB pathway in SAA-stimulated neutrophils. Phosphorylation of NF-κB was induced in neutrophils by SAA stimulation. However, HCQ pretreatment had no influence on this SAA-induced NF-κB phosphorylation (Fig. 5). We then checked the protein expression of NLRP3, a major component of the inflammasome complex, in human neutrophils. Unstimulated neutrophils exhibited marginal expression of NLRP3, but the expression was enhanced in response to SAA stimulation. HCQ pretreatment did not affect the NLRP3 protein expression in SAA-stimulated neutrophils (Fig. 6).

**Fig. 7:**
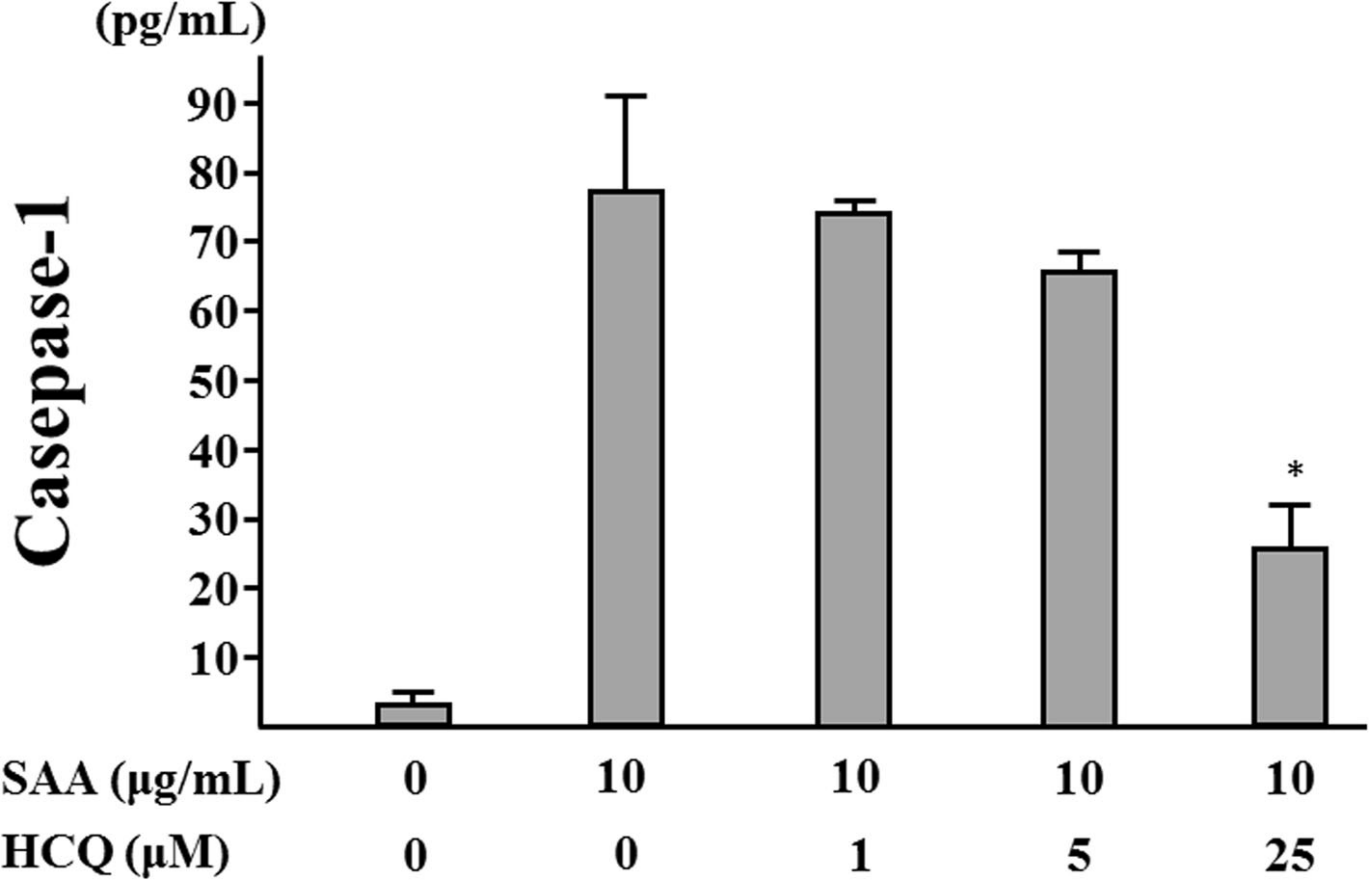
Hydroxychloroquine inhibits the caspase-1 (p20) release from SAA-stimulated neutrophils. Neutrophils were pretreated with the indicated concentrations of hydroxychloroquine for 1 h and stimulated with SAA (10 μg/ml) for 24 h, and supernatants were analyzed for caspase-1 (p20) by ELISA. Values represent the mean ± SD of two independent experiments. *p < 0.05 compared to SAA-stimulated neutrophils

**Fig. 8:**
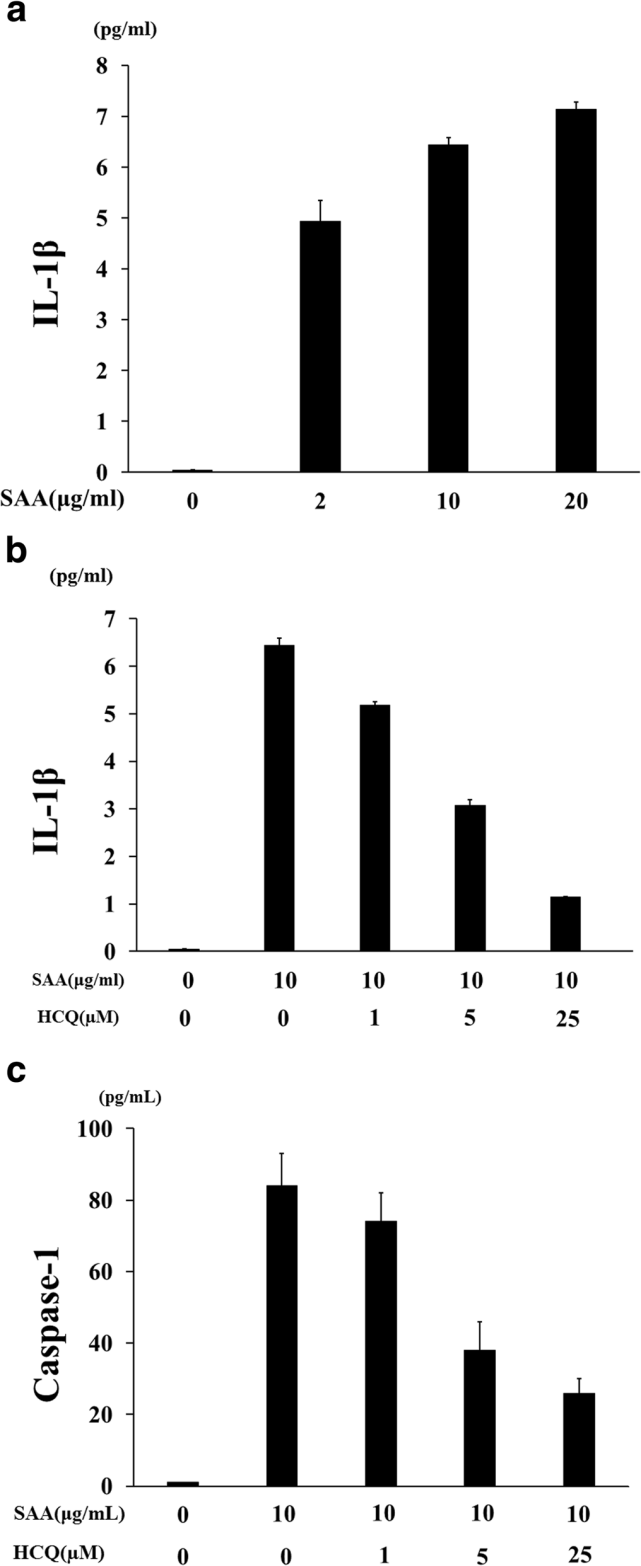
Hydroxychloroquine inhibits IL-1β and caspase-1 (p20) release from SAA-stimulated neutrophils. Neutrophils (2 × 10^6^/ml) were incubated with the indicated concentrations of SAA for 12 h, and supernatants were analyzed for IL-1β production by ELISA (**a**). Neutrophils were pretreated with the indicated concentrations of hydroxychloroquine for 1 h and stimulated with SAA (10 μg/ml) for 12 h, and supernatants were analyzed for IL-1β (**b**) or caspase-1 (**c**; p20) by ELISA. Two experiments were performed using different neutrophils, and a representative result is shown

It is generally accepted that the cleaved form of caspase-1, p20, is released along with processed IL-1β during NLRP3 inflammasome activation [16]. Therefore, culture supernatants were analyzed for secretion of cleaved caspase-1 by an ELISA specific for caspase-1 (p20). Consistent with the impaired IL-1β production, caspase-1 (p20) production was suppressed in neutrophils stimulated with SAA after pretreatment with HCQ in a dose-dependent manner (Fig. 7). Finally, to further confirm the biologic significance of the inhibitory effects of HCQ, we assessed SAA-induced IL-1β or cleaved caspase-1 (p20) secretion from neutrophils at the early time point. To minimize the apoptosis or pyrotosis induction compared to the inflammasome activation in neutrophils, we performed the same assay at an early time point (12 h). SAA stimulation induced IL-1β secretion from neutrophils in a dose-dependent manner even in the short duration of culture periods (Fig. 8a). HCQ pretreatment inhibited SAA-induced IL-1β (Fig. 8b) or caspase-1 (Fig. 8c) secretion from neutrophils in this short duration of culture periods.

## Discussion

Chloroquine and its analog HCQ, originally antimalarial drugs, are widely used for the treatment of rheumatic diseases because of their anti-inflammatory and immunosuppressive effects [1]. However, their effects on immunity and potential mechanisms remain unclear. Previous studies demonstrated that HCQ interferes with the TLR4 signaling pathway and reduces the production of cytokines in LPS-stimulated macrophages [17]. The diverse effects of HCQ on pattern-recognition receptor signaling suggest that the inflammasome could be a target for HCQ [18, 19]. Emerging evidence has suggested an important role for the NLRP3 inflammasome in the pathogenesis of rheumatic diseases [20]. In addition to classical immune cells, activation of NLRP3 was demonstrated in neutrophil-mediated inflammatory processes [21]. In this study, we found that HCQ inhibited SAA-induced IL-1β production in human neutrophils. An exciting clinical implication of the present findings is the identification of HCQ as a potent modulator of autoinflammation by affecting the NLRP3 inflammasome.

Activation of the NLRP3 inflammasome requires two steps that are controlled by different mechanisms [22]. In the first step, NLRP3 expression is induced, and in the second step, NLRP3 activation leads to caspase-1 activation and subsequent pro-IL-1β processing [23]. As a key mediator of immunity, NF-κB plays a critical role for priming of the NLRP3 inflammasome [24]. The NF-κB pathway is also involved in the transcription of pro-IL-1β and NLRP3 expression, which are the limiting steps for NLRP3 inflammasome activation [25].

We found that SAA stimulation led to expression of both pro-IL-1β mRNA and NLRP3 protein. In addition, NF-κB activation was required for NLRP3 protein induction. HCQ pretreatment did not affect SAA-induced NF-κB signaling, a known activator of NLRP3, suggesting that HCQ did not inhibit the priming of the NLRP3 inflammasome. Attenuated NF-κB activation may result in reduced SAA-induced NLRP3 protein expression or pro-IL-1β mRNA expression. However, our data clearly indicated that HCQ pretreatment did not affect SAA-induced pro-IL-1β mRNA or NLRP3 protein expressions. Therefore, HCQ appears to inhibit NLRP3 inflammasome activation by affecting the SAA-mediated NLRP3 activation steps without affecting priming steps.

We showed that HCQ inhibits SAA-induced IL-1β secretion and cleaved caspase-1 (p20) secretion from human neutrophils. These findings suggest that HCQ inhibits SAA-mediated proinflammatory properties by inhibiting the NLRP3 inflammasome activation process. Previous studies demonstrated that stress-induced proteins, including amyloid protein, activate the NLRP3 inflammasome [8]. Consistent with previous reports, our data in human neutrophils indicated that NLRP3 inflammasome activation was induced in amyloid-stimulated neutrophils. We also demonstrated that HCQ limits danger signal-induced IL-1β release from human neutrophils. However, the mechanism by which HCQ exerts its inhibitory effects on inflammasome activation is still incompletely understood.

Because of the diverse effects of HCQ on pattern-recognition receptors and lysosomal function, it is tempting to speculate that the inflammatome could be a target for HCQ during innate immune cell activation. Our data indicated that HCQ may have a protective effect on amyloid-mediated inflammatory processes at the level of neutrophils, as representative innate immune cells. However, it remains unclear how HCQ mediates this inhibition. Recent studies demonstrated that ROS can induce lysosomal damage, leading to enhanced NLRP3 inflammasome activation [26]. HCQ was shown to inhibit ROS production in activated macrophages [27]. Thus, it is possible that HCQ modulates the NLRP3 inflammasome activation process by affecting mitochondrial ROS accumulation.

There is a limitation to this study. Although the HCQ concentrations used in this study were higher than those measured in serum from patients on regimens used to treat SLE or RA, the maximum serum levels we could achieve were 5–10 μM. However, it was reported that the use of higher concentrations of chloroquine for treatment of leukocytes in vitro resulted in intracellular concentrations comparable to those obtained in vivo during chloroquine therapy [28].

## Conclusions

We have demonstrated that HCQ can inhibit SAA-induced IL-1β secretion from human neutrophils. Our results suggest that HCQ may inhibit NLRP3 inflammasome activation by affecting the activation steps, rather than the priming steps. These findings provide insights into the novel anti-inflammatory mechanisms of HCQ and suggest a new strategy for targeting autoinflammatory disorders.

## Data Availability

Not applicable

## Abbreviations

HCQ: Hydroxychloroquine
IL-1: Interleukin-1
NLRP3: NLR family pyrin domain containing 3
SAA: Serum amyloid A
TLR: Toll-like receptor
